# Academic children’s hospital partnership with public health to address mass pediatric community tuberculosis exposure

**DOI:** 10.1017/ash.2025.10040

**Published:** 2025-06-12

**Authors:** Alice I Sato, Bradford Becken, Arthur J Chang, Shirley F Delair, Lourdes Eguiguren, Andrea Green Hines, Clayton Mowrer, Gwenn L. Skar, Jennifer Zwiener, Kari Neemann

**Affiliations:** 1 Children’s Nebraska, Omaha, NE, USA; 2 University of Nebraska Medical Center, Omaha, NE, USA; 3 Douglas County Health Department, Omaha, NE, USA

## Abstract

**Objective:**

To illustrate how a partnership between an academic medical center and a public health department successfully responded to a large tuberculosis (TB) exposure at a community daycare center.

**Setting:**

A multidisciplinary team rapidly established a dedicated TB Exposure Clinic to evaluate and screen exposed children requiring window prophylaxis.

**Patients:**

The exposure affected 592 individuals, including 359 children under five—those at highest risk for severe disease.

**Interventions:**

Given the vulnerability of young children to TB infection, timely evaluation and initiation of window prophylaxis were prioritized.

**Results:**

Over two days, 162 children were assessed for TB window prophylaxis, and 110 additional children underwent TB screening.

**Conclusions:**

By leveraging clinical expertise, interdisciplinary collaboration, and informatics infrastructure, the TB Exposure Clinic delivered rapid, comprehensive care while minimizing disruption to local healthcare systems. This model underscores the essential role of academic medical centers in supporting public health responses.

## Introduction

Tuberculosis (TB), caused by the bacillus *Mycobacterium tuberculosis*, remains a leading global health concern.^1^ Briefly surpassed by COVID-19, tuberculosis has reemerged as the leading infectious disease killer.^1^ It is estimated that 15 million children worldwide are exposed to TB annually, with 1.3 million children under the age of 15 developing disease in 2023.^1^ In the World Health Organization (WHO) Region of the Americas, new infections have steadily risen since a nadir in 2019.^1^ Many TB infections are either eliminated or contained by the body’s immune defenses, remaining subclinical (TB infection, previously termed latent TB). However, some infections progress with viable bacilli multiplying and causing TB disease (previously termed active TB).

It is critical to assess and test individuals exposed to contagious TB. A thorough assessment is necessary, including a physical exam, chest imaging if respiratory symptoms are present, and a tuberculin skin test (TST) or interferon-gamma release assay (IGRA) testing.^2^ Children under five are particularly vulnerable to poor outcomes. A recent study reported a 20% two-year cumulative risk of developing TB disease following exposure, and meningitis or miliary disease are more likely in children <2.^3–5^ Unfortunately, children under 5 years old with TB infection may have a negative TST or IGRA due to anergy or a delayed immune response. In such cases, presumptive TB infection treatment (“window prophylaxis”) should begin to mitigate risk of disease progression, with repeat testing performed 8–10 weeks after exposure to confirm the absence of infection.

TB outbreaks present a significant threat to public health, requiring rapid identification of cases to ensure timely care and prevent spread. However, conducting epidemiologic investigations, performing diagnostic tests, and implementing treatment strategies is challenging, especially in pediatric TB cases. Key components of TB prevention, control, and elimination, as outlined by the Advisory Council for the Elimination of TB and the National Tuberculosis Coalition of America (NTCA), demand substantial resources. Unfortunately, public health funding is often inconsistent and insufficient, exacerbating these challenges.^1,2^ Particularly in low-incidence states where public health responses are hindered by personnel shortages, resource limitations, barriers to healthcare access, and follow-up.^3^

Collaborative efforts among state and local health departments, healthcare systems, and community partners are crucial to strengthening responses to TB outbreaks.^4^ By pooling resources, expertise, and data, these partnerships can more effectively address public health challenges. Herein we describe our children’s hospital experience in assisting the county and state health departments respond to an urgent public health threat.

### Case presentation

In November 2023, the Douglas County Health Department (DCHD), while conducting contact tracing of a smear-positive individual recently hospitalized with pulmonary tuberculosis, found that the affected individual was associated with a drop-in YMCA daycare center. This individual had been symptomatic for 3 months prior to their hospital admission. As the exact onset of the infectious period couldn’t be precisely determined, per guidelines the potential infectious window was set at 3 months before symptom onset, resulting in a 6-month possible exposure period.^6^

DCHD, in collaboration with the Centers for Disease Control’s (CDC) Division of Tuberculosis Elimination, defined a close contact as anyone with a contact period of ≥ 30 minutes. Using an electronic badge system that tracked the entry and exit of each child, health officials quickly identified those with direct exposure to the source patient and calculated the total duration of exposure. Out of 592 identified exposures, 359 involved children under 5 years old, and 211 of those had been exposed within the previous 10 weeks, necessitating evaluation for TB window prophylaxis (see companion article).

**Figure 1.**
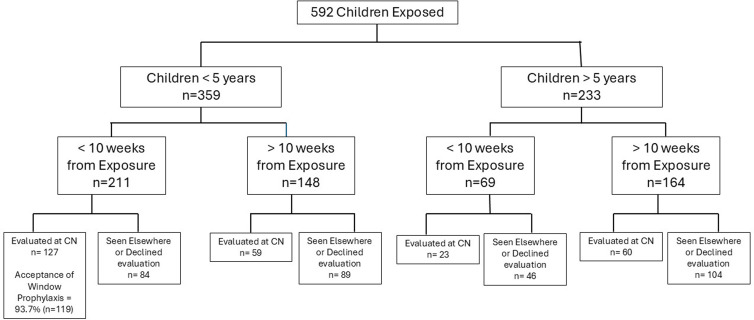
Timeline of events from notification of exposure event to communication of initial testing results to patients and DCHD. CN= Children’s Nebraska, DCHD = Douglas County Health Department, NHHS = Nebraska Health & Human Services, CDC = Centers for Disease Control & Prevention, IT=Information Technology, YMCA = Young Men’s Christian Association, ID = Infectious Diseases, HAN = Health Alert Network communication, TB = Tuberculosis.

Given the large number of young children requiring timely screening, evaluation, and possible prophylactic treatment, DCHD partnered with Children’s Nebraska (CN) to coordinate a swift community response. Within 36 hours of the notification, we mobilized a massive effort to set up a TB Exposure Clinic that over 2 days evaluated 162 children for TB window prophylaxis and provided TB screening to an additional 110 children (Figure 1).

## TB exposure clinic

### Incident leadership

**Figure 2.**
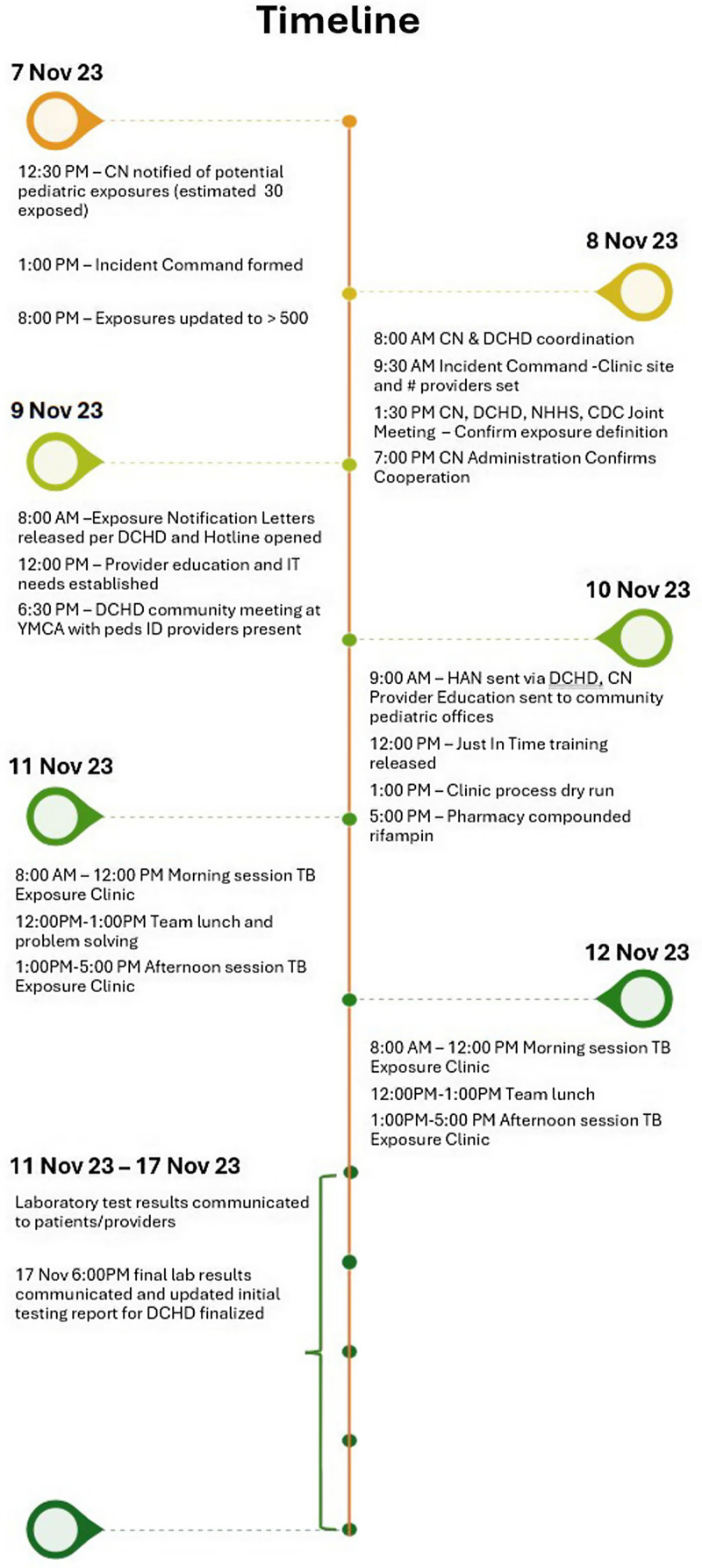
Bar graph of Children’s Nebraska personnel participating in response by job role.

On November 7th, 2023, the Division of Pediatric Infectious Diseases (ID) at CN was alerted to a tuberculosis exposure, initially affecting an estimated 20–30 children (Figure 2). Recognizing the impracticality of fitting this volume of patients into the existing clinic schedule, a leadership team was assembled to address the situation. This initial team included pediatric ID providers, the hospital epidemiologist, and the infection prevention team.

Later that evening, the full extent of the exposure became clear, and by the morning of November 8th, the DCHD confirmed that 211 children required evaluation for TB window prophylaxis. Additionally, CN was asked to serve as an auxiliary TB screening site, supplementing clinics being organized by DCHD.

### Facilities

To set up a clinic with real-time chest X-ray capability and rapid expert interpretation, we considered several locations, including the Specialty Care Center at CN and two Urgent Care locations. In the end, we chose the Specialty Care Center because it had 2 X-ray-capable rooms from the outpatient Orthopedics clinic in continuity with the ID clinic, which had a negative airflow room for patients with pulmonary symptoms.

This location had additional advantages. It did not operate on weekends, so there would be no mixing of exposed patients with other clinic or Urgent Care patients. While part of the CN campus, it is in a separate building from inpatient facilities. It was a geographically convenient location for many families with ample parking. The clinic ran smoothly on one floor, with a central check-in area, multiple hallways of patient rooms, and several workstations for staff.

#### Clinic operation

Within the DCHD notification of exposure letter sent to all affected patients/families were instructions on how to schedule appointments for the evaluation clinic, including a dedicated phone number set up for scheduling and answering basic medical questions. Once an appointment was made, guardians received directions to the clinic, along with parking and arrival instructions.

After appointments were scheduled, ID physicians categorized patients into two groups: Tier 1 and Tier 2. Tier 1 included children under 4 years old who would be eligible for window prophylaxis based on last documented exposure. Tier 2 included children over 4 years or those whose last exposure was more than 10 weeks prior, as they did not require window prophylaxis. TB testing was ordered for both groups (Quantiferon for those 2 years and older, PPD for those under 2 based on 2021–2024 RedBook guidance), and chest X-rays were pre-ordered for all Tier 1 patients.^7^ Orders were thereby easily released at time of patient check-in so that patient flow proceeded efficiently.

On the day of the appointment, patients were met by a greeter at the parking entrance who provided final parking and arrival instructions, including masking of patients and family members, and symptom screening per usual protocol. After check-in at the clinic, medical assistants placed patients in rooms. Initial symptom screening did not require airborne precautions for any patients. Tier 1 patients had their weight and temperature taken, and the nurse reviewed their medical history, allergies, current medications, and last exposure date per guardian’s knowledge. Tier 1 patients were then taken for a chest X-ray. For Tier 2 patients, the nurse reviewed their history, and TB tests were performed in the clinic room. Tier 2 patients were discharged with follow-up instructions. Tier 1 patients were evaluated by a medical provider, who reviewed the chest X-ray and the pediatric radiologist’s real-time interpretation. The provider performed a physical exam and ordered further tests if necessary, such as additional imaging for bone pain. If the family agreed to window prophylaxis, medication and counseling was provided prior to discharge. All patients received instructions on reading their TST if needed, re-testing recommendations, and signs or symptoms to watch for.

#### Diagnostics

To meet the high demand and ensure a faster turnaround the Nebraska Public Health Laboratory (NPHL) agreed to perform expedited IGRAs obtained at both our clinic and additional DCHD response clinics. For children under 2 years old, TST tests were administered during the visit and given detailed instructions on where and when to have them read, either at CN or at one of the DCHD mobile clinics scheduled the following week. IGRA results were typically available within 24–48 hours and the Pediatric ID team promptly communicated the findings to families, either by phone or through the electronic medical record (EMR). All TB testing results were returned and directly communicated to families by the Pediatric ID office nurses and physicians. Results were also sent to all primary care physicians by the end of the business week (within 6 days of visit).

#### Informatics

To ensure efficient tracking of patient testing, prophylaxis, and follow-up, we used an Excel spreadsheet stored on a shared drive accessible to both the ID division and DCHD. We collaborated with our Information Technology (IT) and EMR teams to streamline the HIPAA-compliant process by creating scheduling and note templates, dot phrases, pre-populated lab and imaging orders, primary care provider message templates, family education materials, and standardized window prophylaxis medication orders for our volunteer providers.

All lab orders were placed by a pediatric ID physician, ensuring that results were directed only to the ID division for follow-up, avoiding the need for non-ID providers to manage them. We conducted a technology dry run ahead of the clinic days to test informatics workflows, including scheduling, check-in, rooming, ordering, and prescribing, which helped us identify and resolve a few issues.

One of the pediatric ID physicians managed the spreadsheet, while division members regularly updated it with re-testing lab results as they became available. These updates were shared with the health department.

#### Pharmacy

Communication between the pharmacy and multidisciplinary teams was essential throughout the clinic’s planning and operation. Within 2 hours of mass exposure notification from DCHD the ID pharmacist worked with pharmacy leadership to acquire medication and discuss pharmacy’s role. The pharmacy team (the ID pharmacist, Director of Pharmacy, and Outpatient Pharmacy Manager) participated in discussions about the clinic’s planning and operation. Pharmacy’s early involvement ensured access to medications, equipment, and staff for efficient patient flow.

The outpatient pharmacy agreed to open during the clinic allowing for compounded medication (ie. rifampin) to be rapidly delivered to patients. Pharmacy performed the following prior to day of clinic: scheduled clinic patients were entered into the pharmacy patient database (including insurance information), supplies ordered (medication, bottles, labels, mortars and pestles, flavoring, oral syringes), medication preparation, staffing plans established, and pharmacy workflows developed. The ID pharmacist created educational documents on rifampin and isoniazid for clinic providers and pharmacists that included weight-ranged dosing and counseling. During the clinic, a pharmacist was stationed in the clinic to coordinate efforts with the clinic team and the outpatient pharmacy either in person, phone, or secured text messaging. To ensure all steps were completed a board was used to track when a prescription was entered, patient counseled, and patient received medication and was ready for discharge. Counseling occurred in the clinic room by a pharmacist, pharmacy technician, pharmacy intern, or provider. Most prescriptions were delivered to the clinic, except when payment was required, in which case patients were escorted to the outpatient pharmacy.

#### Personnel

**Figure 3.**
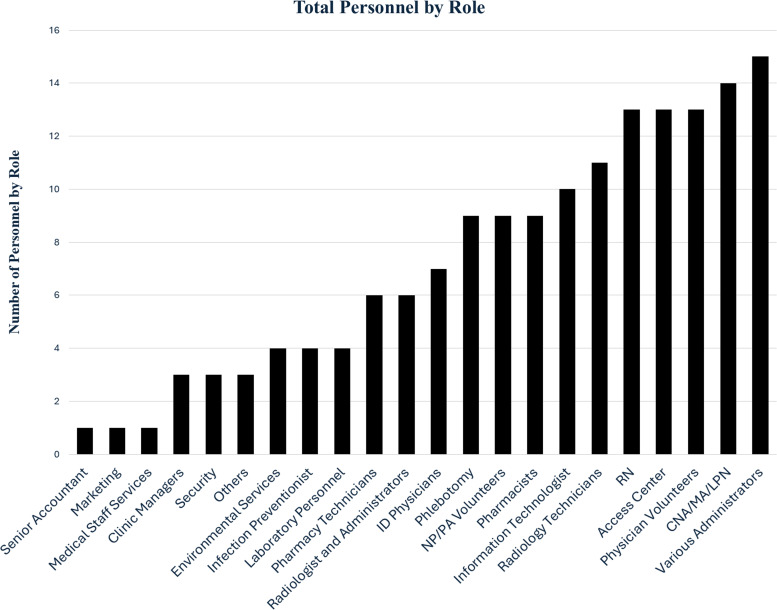
Team members involved in the planning, development, and implementation of the TB Exposure Clinic.

A total of 163 individuals came together to create and operate the clinic, each playing a vital role in its success (Figure 3). From the initial planning stages to final operation, their collaboration ensured a smooth and efficient patient experience. The Access Center coordinated scheduling and served as an information hotline for the community. Administrators oversaw logistics, environmental services maintained a clean and safe space, and information technologists integrated the clinic into the electronic health record system, streamlining workflows. Infection preventionists implemented safety protocols, while laboratory technicians and phlebotomists facilitated on-site testing. Nursing played a crucial role in facilitating patient check-in, reviewing medical history, medications, and allergies, and ensuring a smooth flow of care. They also conducted Tier 2 evaluations for patients requiring only tuberculosis testing, streamlining the process and helping maintain clinic efficiency. Pharmacists and pharmacy technicians prepared and delivered medications while educating patients on their use. Volunteer physicians, nurse practitioners, and physician assistants staffed the Tier 1 portion of the clinic, supported by infectious disease specialists who provided expert guidance in patient evaluations. Radiologic technologists conducted chest X-rays, which radiologists reviewed in real time. Security personnel ensured a safe environment for both staff and patients. Through this coordinated effort, the clinic operated efficiently, seeing a high volume of patients with minimal delays in care. Volunteers were solicited through internal e-mail and time was compensated.

### Education

To meet the patient demand, we determined that 24 half-day clinics (ie. 6 providers per session over 2 d) were necessary. As this demand exceeded the staffing capabilities of the Pediatric ID division, non-ID pediatric providers were enlisted. Therefore “Just in Time” training was provided on clinic operation and education on pediatric tuberculosis. This included information addressing common patient and family questions, such as tuberculosis epidemiology, its presentation in children (including extrapulmonary complications), necessity of window prophylaxis, and an overview of required medications for both window prophylaxis and TB infection treatment. To address complex questions and evaluate potential tuberculosis cases, board-certified pediatric ID specialists were available during all shifts for real-time consultations and in-person assessments.

### Cost

CN covered upfront costs associated with setting up the clinic pending patient billing and insurance reimbursement. Providers, including ID physicians, volunteer providers, radiologist, and clinical staff generated $24,900 in costs related to stipends. Providers were paid a flat fee per half-day shift, regardless of their primary salary or RVU-based compensation. Non-clinician staff generated an additional $12,600 in costs. Given the number of patients planned for the clinic additional medication and pharmacy supplies were ordered, costing $5,367, leading to total upfront costs of $42,867. The clinic evaluated 272 patients, leading to an upfront cost-per-patient of $157.60. Not included in the clinic’s costs were the efforts salaried members of Pediatric ID division, facilities management, pharmacy, and infection preventionists who redirected their efforts in the week leading up to the clinic to create protocols, create just-in-time education materials, and generating an operations plan, while coordinating these efforts with the DCHD. Also not included was the time of pediatric infectious disease physicians and nursing staff spent providing families, their primary care providers and the DCHD with updates as laboratory testing results returned or sending reminders to families when re-testing was indicated. All clinic costs were recuperated through normal patient mechanisms.

## Discussion

Academic medical centers (AMCs) are uniquely situated to partner with public health and provide expertise, resources and access to specialized medical professionals beyond those within local and national public health organizations. Partnerships may be leveraged for both short-term and long-term needs. These partnerships may take many forms. From deployment of mobile medical teams from AMCs to support outbreak investigations to systemic partnerships to provide ongoing clinical services and public health employees leased to the local health department from a hosting university.^8–10^

AMCs and public health partnership allow for improved contact tracing and case identification, enhanced data transfer, increased diagnostic capacity, trust building with communities, and the integrated innovative use of technology such as setting EMR best-practice alerts.^10^

Here we report our experience in leading a children’s health system and AMC response to a large community exposure to tuberculosis with many young children involved. Due to longstanding close working relationships between our infectious disease and infection prevention teams and public health officials at the county and state level, we assisted in leading the community response to this exposure. We rapidly established a multidisciplinary response team (mimicking institutional incident command structures) within CN to bring all stakeholders together for planning and execution of our portion of the response.

Our primary goal was to ensure that the children in our communities were able to be rapidly assessed, screened and offered appropriate care that was simple for families to navigate. Not all exposed patients receive their primary care through CN clinics and our intent was not to replace their own providers. We collaborated with the DCHD to provide communication and guidance to all regional providers. Additionally, our outpatient pharmacy was able to fulfill prescriptions (including compounded rifampin) to patients seen elsewhere, as compounding is not readily available in our area. In setting up our clinic, we prioritized the youngest children who needed window prophylaxis, but allowed for other children to be seen, including exposed older siblings. Nebraska DHHS was able to procure funding for the care of uninsured children affected by this exposure, and no children were turned away based on inability to pay. Patients benefited from being able to receive all components of care at a single comprehensive visit. Complicated cases were seen in real time by pediatric infectious disease providers. Instructions and printed orders for retesting were provided if indicated. Documentation of the visit and follow-up recommendations were transmitted directly to primary care clinics for further management with the ability to refer back to Pediatric ID if desired. CN had upfront costs as described above. However, costs were recuperated through standard medical billing.

In addition to increased efficiency and quality of care for patients, the specialized clinic minimized disruptions to healthcare operations across multiple settings. Individual evaluations spread over multiple sites (clinics, laboratories, and radiology) could have created fear and uncertainty resulting in additional infection prevention burden. Secondly, information was consistently and rapidly shared with patients’ primary care providers and with public health officials, which likely reduced the number lost to follow up and adverse events. The success of this public health and AMC partnership was recognized by the DCHD Board of Health and patient experience scores were overwhelmingly positive. We hope that sharing our experience will assist other AMCs in supporting their public health departments with swift and effective responses to events involving widespread community exposures.

## Research Transparency and Reproducibility

NA
